# Cervico-Vaginal Immunoglobulin G Levels Increase Post-Ovulation Independently of Neutrophils

**DOI:** 10.1371/journal.pone.0114824

**Published:** 2014-12-05

**Authors:** Katrein Schaefer, Najmeeyah Brown, Paul M. Kaye, Charles J. Lacey

**Affiliations:** Centre for Immunology and Infection, Hull York Medical School and Department of Biology, University of York, Heslington, York, United Kingdom; The Hospital for Sick Children and The University of Toronto, Canada

## Abstract

The prevalence of sexually transmitted infections (STIs) is often higher in females than in males. Although the reproductive cycle profoundly modulates local immunity in the female reproductive tract (FRT) system, significant gaps in our knowledge of the immunobiology of the FRT still exist. An intriguing and frequently observed characteristic of the FRT is the predominant presence of immunoglobulin (Ig) G in cervico-vaginal secretions. We show here that in the mouse, IgG accumulation was enhanced approximately 5-fold post-ovulation, and was accompanied by an influx of neutrophils into the FRT. To determine whether these two events were causally related, we performed short-term neutrophil depletion experiments at individual stages throughout the estrous cycle. Our results demonstrate that neutrophils were not necessary for cycle-dependent tissue remodeling and cycle progression and that cycle-dependent IgG accumulation occurred independent of neutrophils. We thus conclude that neutrophil influx and IgG accumulation are independent events that occur in the FRT during the reproductive cycle.

## Introduction

STIs are a major public health problem worldwide. In many settings the incidence and prevalence of STIs are higher in females than in males. For example, in sub-Saharan Africa 57% of individuals infected with human immunodeficiency virus (HIV) are women [1], and 70% of the herpes simplex virus 2 (HSV-2)-infected population are female [2]. Similar trends have been observed with bacterial STIs, such as those caused by *Chlamydia trachomatis* [3]. As well as socio-demographic factors, such as age, behavior, hormonal contraception and partnership characteristics, which can impact on the incidence of STIs [3], it is generally believed that an innate biological susceptibility lies behind the observed excess of STI acquisition in females. One of the most obvious gender differences is the female reproductive cycle, which is determined by the cyclically changing concentrations of the ovarian female sex hormones estradiol and progesterone [4]. These sex hormones modulate the local immune system in the FRT, including the regulation of immunoglobulins (Igs) in cervico-vaginal secretions [5]–[9] and differential abundance and function of myeloid cells within the tissue [10], [11]. It is therefore possible that physiological changes in the FRT, caused by sex hormones, can make females more susceptible to acquiring STIs at certain time-points during the reproductive cycle.

An unusual feature of the FRT as a mucosal site is that IgG is the predominant antibody isotype in reproductive secretions during health and infection [7]–[9], [12], [13], as opposed to other mucosal sites [14]. Moreover, it has been shown that IgG is the main antibody isotype in genital secretions that protects against sexually transmitted pathogens in humans [13], in experimental STI models using macaques [15] and in rodents [16]. Additionally, it has been reported that cervico-vaginal IgG increased following systemic or mucosal vaccination [17]–[20]. These findings strongly suggest a key role for IgG in FRT mucosal defense. Levels of IgG in cervico-vaginal lavage fluid have been shown to be influenced by the reproductive cycle [5]–[9], although these data are not entirely consistent. Whereas some groups have observed increased IgG in reproductive secretions post-ovulation (increasing progesterone levels) [5]–[7], others detected the highest IgG levels prior to ovulation (increasing estrogen levels) [8], [21]. Even though it has been consistently observed that cervico-vaginal IgG levels change throughout the reproductive cycle it is currently not clear where IgG originates from and also how it reaches reproductive secretions. It has been proposed that IgG reaches cervico-vaginal secretions by passive transudation from serum [22], or that it is locally produced by plasma cells [23], [24]. However, neither hypothesis has been convincingly demonstrated to date. Regardless of origin it is possible that IgG is actively transported into cervico-vaginal secretions by a specific transport process [25]. The neonatal Fc receptor (FcRn) is thought to play a major role in IgG transport across mucosal barriers [26], including the FRT [27].

Neutrophils are key players in the innate immune system and rapidly migrate to sites of traumatic injury or acute infection where they contribute to pathogen killing and clearance. FRT neutrophils are known to play a protective role during vaginal infection with HSV-2 [28], [29], *Neisseria gonorrhoeae* [30], *Trichomonas vaginalis* [31], and *Chlamydia* [32]. Neutrophils are also present in the healthy FRT. In humans, the numbers of vaginal neutrophils remains relatively unchanged throughout the reproductive cycle [33], whereas there is a vast neutrophil influx into the uterus before the onset of menses [34]. This influx may well be associated with secretion of IL-8 by the uterine endometrium prior to menses [35]–[37]. As in the human uterus, there is a significant neutrophil migration into the rodent vagina post-ovulation [38], [39]. This influx is preceded by a surge of MIP-2 (rodent IL-8 homologue) secreted by vaginal epithelial cells during ovulation [40]. Neutrophils in the FRT may be linked to changing IgG levels throughout the reproductive cycle, as they have recently been reported to act as helper cells for B cell activation, including production of IgG [41]. Moreover, neutrophils may also contribute to normal cycle progression, as has been previously suggested [42]. Through the secretion of elastase [43] or collagenase [44], they may be involved in cycle-dependent tissue remodeling, and the battery of mediators produced by neutrophils may have a role in local cycle-dependent immune regulation. However, these questions have not been formally addressed to date.

Here, we investigate further by which mechanism IgG reaches FRT secretions. Due to the overlapping kinetics of cervico-vaginal IgG levels and the neutrophil influx post-ovulation, we have asked whether there is a causative link between these two events. To our knowledge, this is the first study testing this hypothesis. Importantly, we found that IgG content in cervico-vaginal secretions of C57BL/6 mice peaked post-ovulation (increasing progesterone levels), in concert with a pronounced neutrophil influx into the FRT. To determine whether neutrophils played a casual role in IgG secretion, either directly or through tissue remodeling, we developed a model that allowed timed neutrophil depletion. Our results show that cervico-vaginal IgG accumulation is regulated independently of neutrophils. In addition, we detected no role for neutrophils on cycle-dependent tissue remodeling (vaginal epithelial shedding) or cycle progression, contrary to studies using cell depletion regimens that also deplete inflammatory monocytes.

## Results

### Characterization of the estrous cycle of the mouse

**Figure 1:**
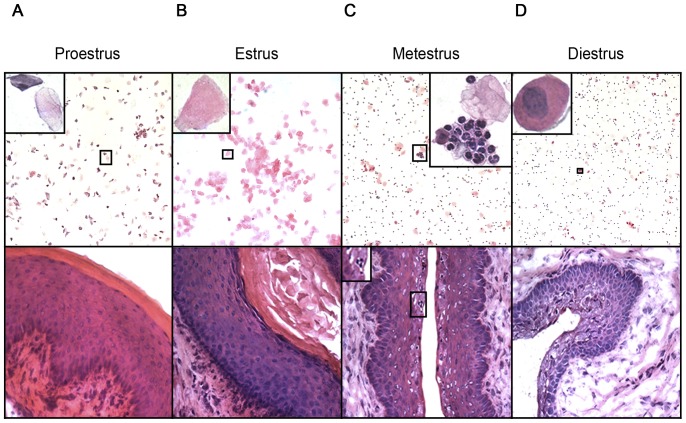
Tissue changes in the FRT during the estrous cycle of the mouse. Vaginal smears were taken from naïve virgin C57BL/6 female mice (8–12 weeks old) over the course of one estrous cycle and used for determination of cycle stage. Mice were killed at PE (**A**), E (**B**), ME (**C**) and DE (**D**) and tissue sections of vagina were cut. Vaginal smears (top panel) and tissue sections (bottom panel) were stained with H&E. The insets in the top panel show typical cell types found in vaginal smears of each cycle stage. The inset in the bottom panel shows neutrophils present in vaginal epithelium. Representative images from conventional cycle stage assessments are shown. Top panel, 100× magnification; bottom panel, 400× magnification.

Estrous cycle stages in naïve virgin 8–12 week-old female C57BL/6 mice were determined from vaginal smears and frozen vaginal tissue sections stained with H&E (**Figure 1**). The murine estrous cycle has four stages; proestrus (PE), estrus (E), metestrus (ME) and diestrus (DE), and one cycle is completed within 4–5 days [38]. Importantly, E is the time during which ovulation occurs (high serum estrogen levels) and DE is the cycle stage during which serum progesterone levels peak [45]. Three dominant cell types can be found in vaginal smears of the mouse, namely cornified epithelial cells (lacking a nucleus), nucleated epithelial cells and leukocytes (in particular neutrophils), and cycle stage is determined by the relative abundance of these cell types in vaginal smears. PE smears consisted of round, nucleated epithelial cells mixed with cornified epithelial cells (**Figure 1A** **, top panel**) which make up the outermost layer of the vaginal epithelium (**Figure 1A** **, bottom panel**). E smears consisted entirely of large cornified cells (**Figure 1B** **, top panel**). The epithelial layer was the thickest during this cycle stage (**Figure 1B** **, bottom panel**). ME smears contained a large number of tightly packed neutrophils (defined by their multilobulated nuclei), which were often attached as cell clumps to epithelial cells (**Figure 1C** **, top panel**) and which were present within vaginal epithelium (**Figure 1B** **, bottom panel**). DE smears consisted mainly of dispersed neutrophils mixed with nucleated epithelial cells. No cornified cells were present in vaginal smears during this cycle stage (**Figure 1D** **, top panel**). The vaginal epithelium was the thinnest during DE (**Figure 1D** **, bottom panel**).

### Endogenous cervico-vaginal IgG concentration peak post-ovulation

**Figure 2:**
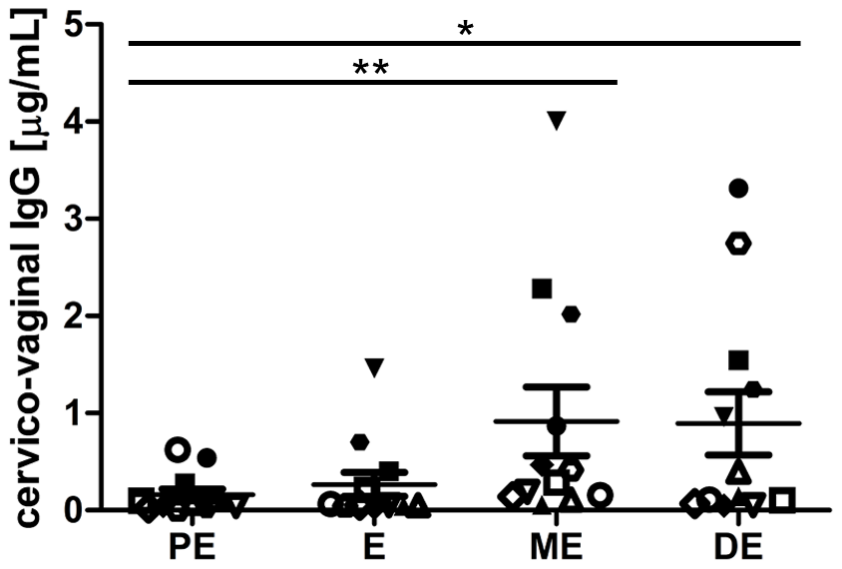
Influence of the estrous cycle on cervico-vaginal IgG levels. Vaginal washings were taken from naïve adult female C57BL/6 mice (8–12 weeks old) throughout the course of the estrous cycle. Samples were analyzed by mouse IgG-specific ELISA. Each graphic symbol represents one experimental animal. Data are derived from three independent experiments (n = 4) and were analyzed using one-way ANOVA with post-test (Tukey's). The mean±SEM is shown (*, p≤0.05; **, p≤0.01).

Endogenous IgG concentration in FRT lavage fluid was analyzed during the course of the estrous cycle. Cervico-vaginal washings were taken from naïve virgin female mice (8–12 weeks old) throughout one complete cycle in a manner that allowed assessment of the amount of IgG that accumulated in FRT secretions during discrete 24h periods (**Figure 2**). IgG accumulation during the 24h period comprising PE was low (0.16±0.05 µg/ml). During E, IgG concentration increased approx. two fold, and was highest during ME (0.9±0.35 µg/ml; p≤0.01 relative to PE). IgG accumulation in cervico-vaginal lavage fluid did not significantly change between ME and DE (0.9±0.33 µg/ml).

### Neutrophils infiltrate the FRT post-ovulation

**Figure 3:**
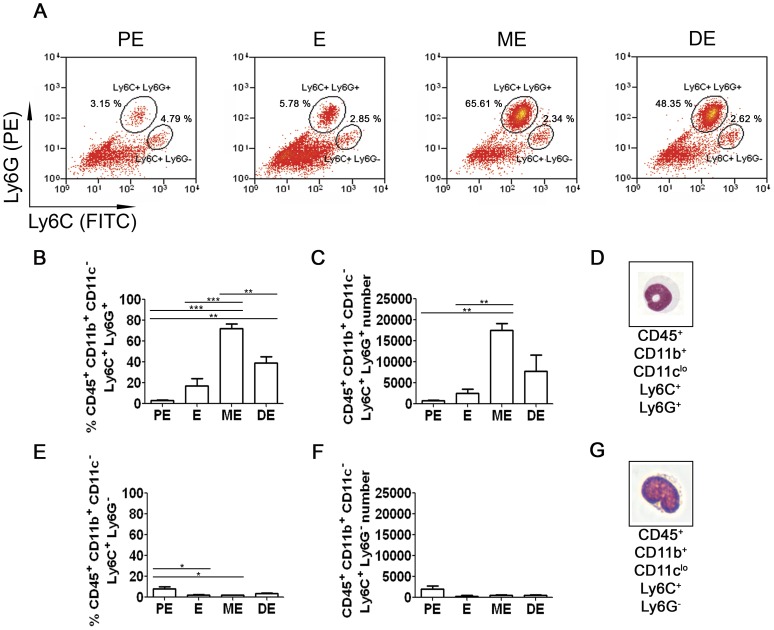
Detection and quantification of neutrophils and monocytes in the total lower FRT during the estrous cycle by flow cytometry. Lower FRT was isolated from naïve adult virgin female C57BL/6 mice and prepared for flow cytometry. Cells were gated as CD45.2^+^CD11b^+^CD11c^lo^ (as shown in **Figure S1**) prior to further analysis. (**A**) Percentage of CD45.2^+^CD11b^+^CD11c^lo^Ly6C^+^Ly6G^+^ cells (neutrophils) and CD45.2^+^CD11b^+^CD11c^lo^Ly6C^+^Ly6G^−^ (monocytes) at each cycle stage (estrus,E; proestrus, PE: metestrus, M; diestrus, DE). (**B–D**) Frequency (**B**), absolute number (**C**) and morphology (**D**) of neutrophils at each cycle stage. (**E–G**) Frequency (**E**), absolute number (**F**) and morphology (**G**) of monocytes at each cycle stage. Samples in **(D)** and **(G)** were Giemsa stained cytospins after sorting. Representative images shown in **(D)** and **(G)** were taken at 400× magnification. Data derived from three independent flow cytometry experiments (each run with tissue pooled from four mice per time point) were quantified and analyzed using one-way ANOVA with Tukey's post-test. The mean±SEM is shown (*, p≤0.05; **, p≤0.01; ***, p≤0.001).

Lower FRT tissue (vagina and cervix) was taken from 8–12 week-old naïve virgin mice and prepared for analysis by flow cytometry and FACS. FRT cell suspensions were stained for the hematopoietic cell marker CD45.2, and for the myeloid cell markers CD11b, CD11c, Ly6C and Ly6G (for gating strategy see **Figure S1**), in order to distinguish neutrophils from monocytes (**Figure 3**). The number and percentage of the Ly6C^+^Ly6G^+^ neutrophils significantly increased from PE to ME (approx. 750 cells/lower FRT vs. 17500 cells/lower FRT, representing approximately 3% vs. 72% of CD45^+^CD11b^+^CD11c^lo^ cells; p≤0.01–0.001) (**Figure 3A**–**C**). The abundance of the Ly6C^+^Ly6G^−^ monocytes did not change significantly during the estrous cycle (**Figure 3A, E and F**). Morphology of the sorted cells confirmed the neutrophil (**Figure 3D**) and monocyte (**Figure 3G**) phenotype.

**Figure 4:**
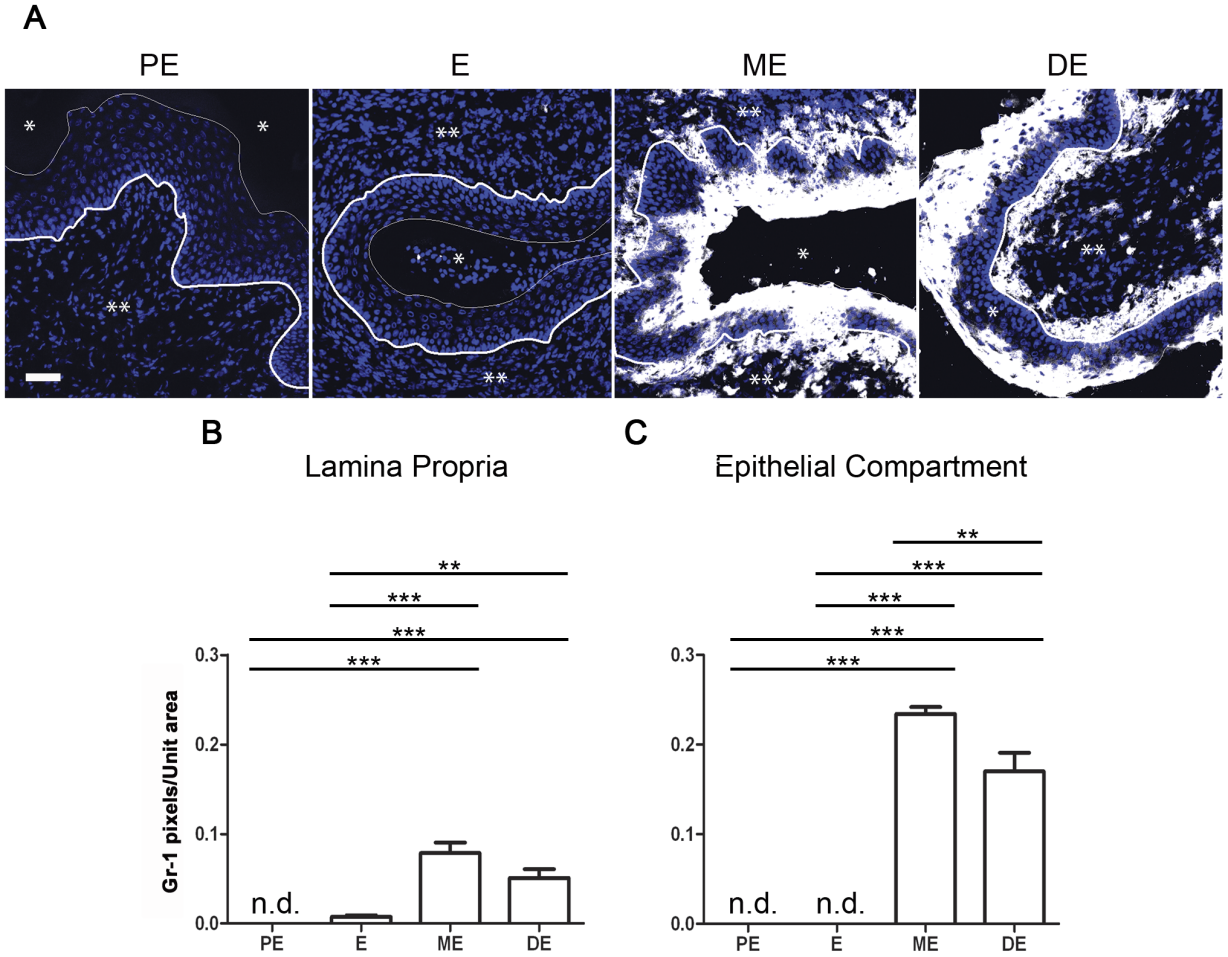
Detection and quantification of Gr-1^+^ neutrophils/monocytes in the vagina during the estrous cycle by immunofluorescence. Tissue was taken from naïve adult virgin female C57BL/6 mice and vaginal tissue sections were stained with DAPI (blue) and mAb RB6-8C5 (Gr-1; white). Representative images from one experiment are shown. The scale bar represents 50 µm. *, lumen; **, lamina propria; thick line, basal membrane; thin line, epithelial cell-lumen border. Data represent a single optical slice (**A**). Images (n = 12) were quantified with the Image J software by pixel count per unit area. Analyses were performed on lamina propria (**B**) and epithelial compartment (**C**) using one-way ANOVA with Tukey's post test. The mean±SEM is shown (**, p≤0.01; ***, p≤0.001; n.d., not detected).

In addition to flow cytometry analyses, the presence of inflammatory granulocyte differentiation factor-1 (Gr-1)^+^ cells [46] in the mouse vaginal epithelium and submucosa was assessed by fluorescence microscopy (**Figure 4**). Gr-1 consists of Ly6G, expressed on neutrophils and transiently on bone marrow monocytes, as well as of Ly6C, expressed on neutrophils and subpopulations of monocytes [47]. No vaginal Gr-1 staining was detected during PE and E (**Figure 4A**). However, Gr-1^+^ cells increased in abundance in the vagina during ME (post-ovulation) and remained high thereafter (p≤0.001 versus PE and E) (**Figure 4A–C**). Furthermore, Gr-1^+^ cells were found in approximately 3-fold higher number in the vaginal epithelium than in the submucosal tissue (**Figure 4B and C**). This data together with flow cytometry, suggest that neutrophil influx into the vaginal epithelium represents the bulk of a cellular inflammatory response during ME and DE.

### IgG accumulation in cervico-vaginal secretions is not affected by neutrophil depletion

**Figure 5:**
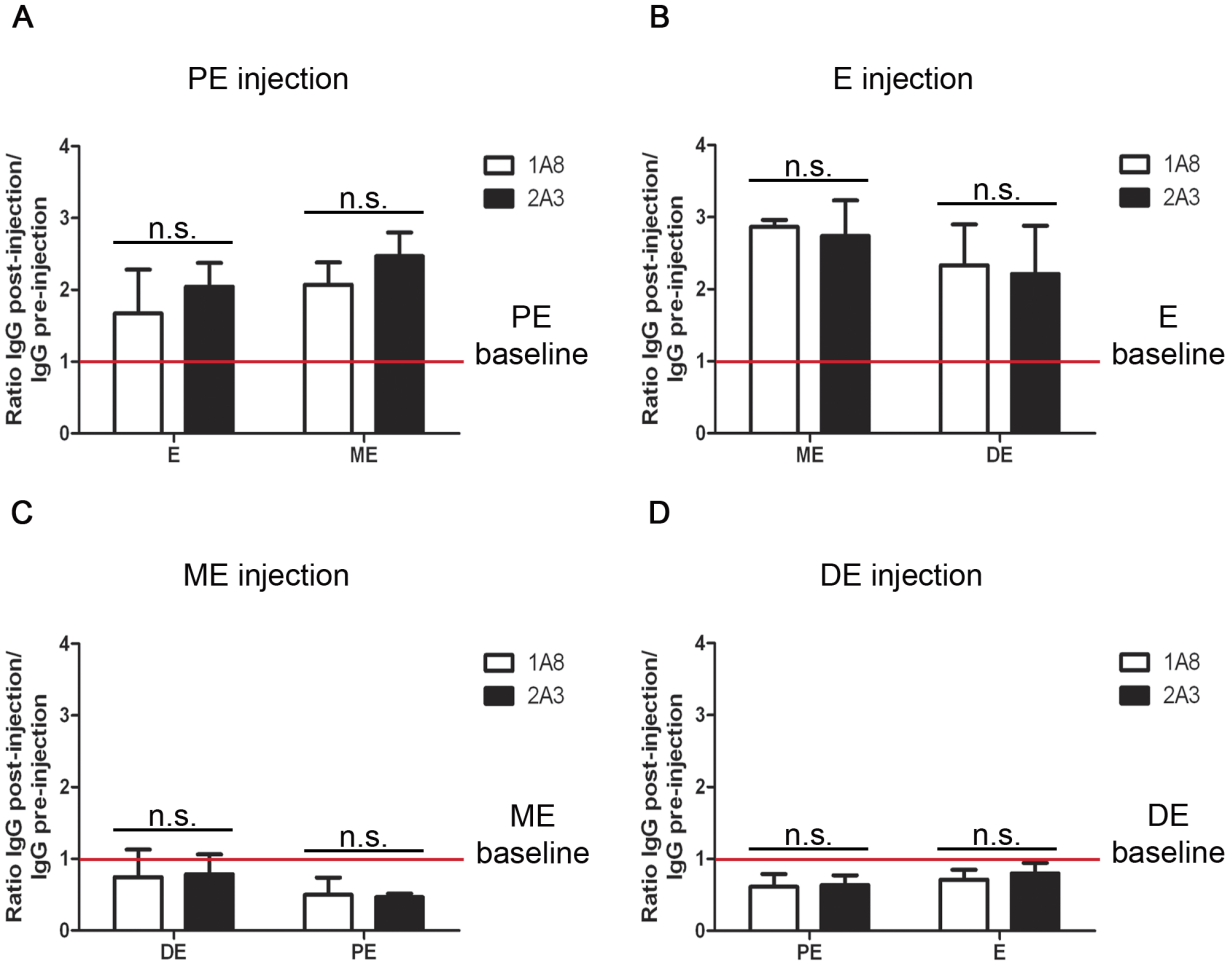
Effects of Ly6G depletion on cervico-vaginal IgG. Naïve virgin C57BL/6 mice were treated with the Ly6G-depleting antibody 1A8 or the isotype control 2A3 at PE (**A**), E (**B**), ME (**C**) or DE (**D**). Cervico-vaginal washings were taken 24 and 48h after mAb administration according to the described protocol. Data are presented as ratios of genital IgG levels post-injection, measured by ELISA, relative to genital IgG levels pre-injection (baseline). Data are derived from one experiment (n≥4 mice) and were analyzed using unpaired t test. The mean±SEM is shown (n.s., non-significant).

The coordinate increase in cervico-vaginal IgG with neutrophil influx into the FRT during ME led us to hypothesize that neutrophils might play a causal role in IgG accumulation in reproductive tract lavage fluid. Thus, we analyzed IgG content in vaginal washings of neutrophil-depleted mice compared to control treated mice. Groups of mice were administered mAb 1A8 at the onset of specific cycle stages and then vaginal secretions sampled 24 h later. The antibody clone 1A8 is known to specifically deplete neutrophils as it targets the surface antigen Ly6G, which is only transiently expressed during monocyte development [47]. Analysis of peripheral blood demonstrated depletion of >90% of neutrophils (**Figure S2A and B**). In addition, frozen vaginal tissue sections taken from 1A8-treated mice were almost devoid of Gr-1^+^ cells (**Figure S2C and D**). In contrast, treatment did not affect abundance of F4/80^+^ cells (**Figure S2C and E**), which is a murine pan-macrophage surface marker. In spite of this highly effective depletion, we found that cervico-vaginal IgG concentration was not significantly different between neutrophil-depleted mice and the control antibody-treated mice (**Figure 5**). Furthermore, IgG concentration in FRT lavage fluid still followed the same trend during the estrous cycle as described above (**Figure 2**). Similar results were also obtained after administration of RB6-8C5 which depletes both neutrophils and monocytes (**data not shown**).

### Neutrophil depletion does not alter estrous cycle progression and vaginal epithelial shedding

**Figure 6:**
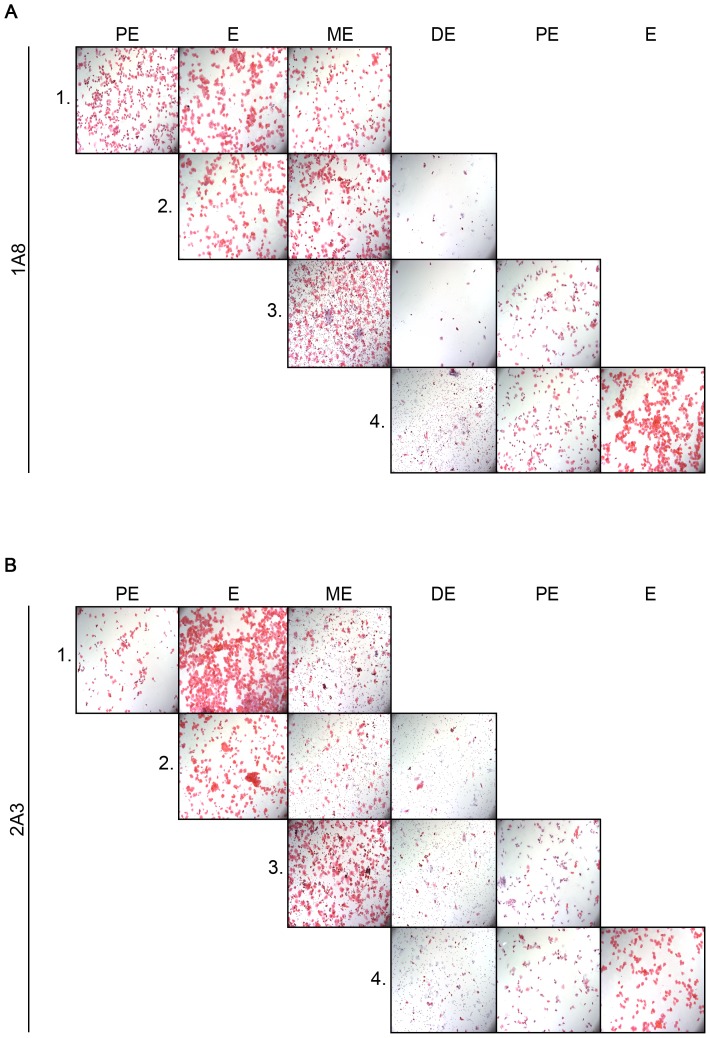
Estrous cycle progression in Ly6G-depleted mice. Four groups of naïve virgin C57BL/6 mice were injected i.p. with 200 µg of mAB 1A8 (**A**) or control mAB 2A3 (**B**). Each group was injected at a different time point during the estrous cycle (group 1 = PE, group 2 = E, group 3 = ME, group 4 = DE). Vaginal smears were taken each day of the experiment and stained with H&E. The smears were used for determination of cycle stage and cycle progression. Representative images from five mice in each experimental group are shown. Images were taken with 100× magnification.

Vaginal smears were used in order to assess cycle progression in neutrophil-depleted and control mice. Cycle progression was not influenced by neutrophil-depletion (**Figure 6**). Neutrophil-depleted mice still cycled irrespective of the cycle stage during which the animals were treated. Vaginal smears from mice treated with 1A8 at ME and DE lacked neutrophils, but appeared normal otherwise (**Figure 6A**). Cycle progressed normally in control antibody-treated mice (**Figure 6B**).

**Figure 7:**
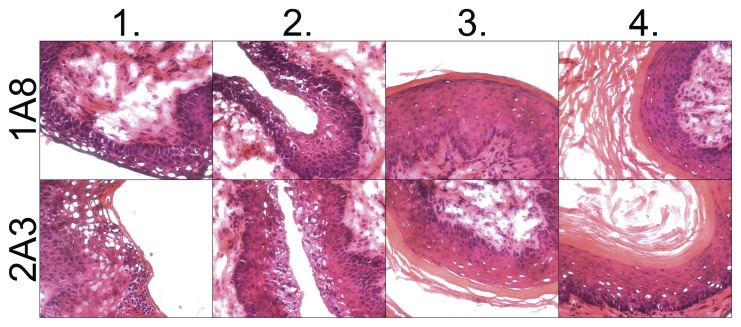
Vaginal epithelial shedding in Ly6G-depleted mice. Four experimental groups of naïve virgin C57BL/6 female mice were injected i.p. with mAb 1A8 (upper panel) or control mAb 2A3 (lower panel). Each mouse group was injected at a different cycle stage. FRT tissue sections were cut and stained with H&E. The tissue sections were used to assess vaginal epithelial shedding in Ly6G-depleted mice. Representative images are shown from five mice in each group. Images were taken at 400× magnification.

Frozen sections of vaginal tissue from animals treated with 1A8 or 2A3 were stained with H&E to analyze whether neutrophils contribute to shedding of vaginal epithelium which occurs from ME to DE. No differences in appearance of the vaginal epithelium were detected during each cycle stage when comparing mice with and without neutrophils (**Figure 7**). Collectively, these data showed that neutrophils do not influence cycle progression or vaginal epithelial cell shedding.

A previous study in which mice were treated over sustained periods with RB6-8C5, which depletes neutrophils, monocytes and other minor populations of Ly6C/G^+^ cells [47], concluded that cycle progression was inhibited following neutrophil depletion [42]. However, using the short term depletion approach described above, we found no evidence that depletion of Gr-1^+^ cells affected cycle progression or vaginal epithelial cell shedding (**data not shown**), consistent with our results using 1A8 to specifically deplete neutrophils.

## Discussion

The immune system of the FRT is regulated in concert with the constantly changing levels of the ovarian female sex hormones estrogen and progesterone, which allow protection of the host from pathogens whilst maintaining the reproductive functions of the tissue [11]. Conflicting data exist regarding hormone-induced immunological changes in the healthy FRT, including altered cervico-vaginal IgG accumulation and differential abundance and function of myeloid immune cells, in particular neutrophils. An important observation made during this work was that genital IgG concentration varied between discrete 24-hour intervals during the four stages of the estrous cycle with significantly elevated IgG concentration during ME (post-ovulation) in C57BL/6 mice. This observation is similar to a previous study in which cervico-vaginal IgG levels were shown to peak during DE [27]. It is not known why IgG concentration is highest post-ovulation (increasing progesterone levels). It has been previously reported that IgG concentration in reproductive mucosal secretions varies during the reproductive cycle in humans [6], [7] and rodents [5] possibly with an inverse correlation between cervico-vaginal IgG concentration and estradiol [9], [21], [48]. Alternatively, FcRn, which has been implicated in IgG transport into the FRT [23] might vary in its abundance or function over the reproductive cycle. Such changes have not been documented, however. Given the protective role of IgG in genital secretions against pathogenic organisms [13], [15], [16], it is tempting to speculate that elevated IgG concentrations post-ovulation could perhaps maximize the chances of successful fertilization and implantation.

Our observations also confirm previous reports of a neutrophil influx into the healthy FRT post-ovulation [39], [40]. It is known that reproductive epithelial cells secrete the potent neutrophil attractant IL-8 and its murine homologue MIP-2 when estrogen levels are elevated [40], [49], [50], providing one explanation for this neutrophil influx into the FRT. Neutrophils migrating into the healthy FRT after ovulation may participate in protection against pathogen invasion fulfilling these functions when vaginal epithelium is being shed and potential pathogens could breach the weakened mucosal barrier. However, recent studies have emphasized the complexity of neutrophil migration and function. For example in *Candida* vaginitis, S100 alarmins are the key neutrophil chemotactic signal, but recruited neutrophils appear not to be the direct mediators of control of the infection [51]. Furthermore, systems biology analyses have recently identified specific and complex molecular networks of the regulation of neutrophil differentiation and chemotaxis in the vagina [52].

As both IgG and neutrophils have host protective function, we asked whether their accumulation may be linked during cycle, with the former dependent upon the latter. Significantly, we have now formally shown that accumulation of cervico-vaginal IgG in a 24h period during each cycle stage was unaffected by neutrophil depletion. IgG concentration still peaked during ME (post-ovulation) and the depletion did not result in a decrease or increase of IgG levels in cervico-vaginal lavage fluid when compared to the control group. Furthermore, neutrophil-depleted mice continued to cycle normally as determined by vaginal smears. In addition, there were no signs of abnormal epithelial cell shedding post-ovulation in neutrophil-depleted mice. Similar observations were made when animals were treated with the Gr-1-depleting antibody RB6-8C5. In a previous study [42] inflammatory cell depletion was performed by repeated administration of the mAb RB6-8C5 and with and end point of 7 days. Using this longer time point, cycle progression was blocked at DE in treated mice, regardless during which cycle stage mAb RB6-8C was administered. Cycle progression re-commenced when neutrophil numbers recovered suggesting that cycle blockade occurred secondary to reduced serum levels of estrogen and progesterone in neutrophil-depleted animals [42]. The reason for the block at DE was not investigated however. Whilst both this and our own study are in agreement that cycle progression is normal two days post-antibody treatment, the broader activity of mAb RB6-8C5 [47] does not rule out a role for inflammatory monocytes in regulating cycle progression. For example, it has been reported that human peripheral CD68^+^ monocytes are responsible for secretion of elevated levels of tumor necrosis factor (TNF)-α just prior to and after ovulation [53], [54], thereby thought to initiate tissue and vascular remodeling within the FRT necessary for menstruation [53]. Importantly, TNF-α has also been described to influence progesterone and estradiol secretion by the ovary, therefore having an important role for ovarian function and ovulation [55], [56].

Another observed feature of mAb 1A8 (or RB6-8C5)-induced neutropenia is its interfering effect on differentiation and function of systemic natural killer (NK) cells [57]. There is no evidence to suggest, however, that NK cells play a crucial role in vaginal clearance of sexually transmitted pathogens [58], [59] or simple hypothesis to explain how NK cells might directly regulate local IgG secretion. Similarly, it has been recently shown that neutrophils provide help for B cell activation and consequently Ig production [41], however, due to the paucity of IgG-secreting plasma cells residing in the FRT [16], [60] this newly identified neutrophil function is also unlikely to explain IgG transport and their changing levels in FRT secretions.

In conclusion, we have shown that, although IgG and neutrophils are coordinately regulated through the estrous cycle, short-term neutrophil depletion neither affects IgG accumulation in reproductive secretions, nor vaginal epithelial remodeling. Our study therefore excludes that IgG transport into reproductive secretions is regulated by neutrophils providing further insight into elucidating this fundamental question. Thus the function of neutrophils in the healthy FRT requires further elucidation, as the cyclic migration and infiltration of these cells indicate a crucial functional role within the FRT.

## Methods

### Ethics statement

All animal care and experimental procedures were performed under UK Home Office License (Ref # PPL 70/5611) and with approval from the Animal Procedures and Ethics Committee of the Department of Biology, University of York.

### Animals and tissue samples

Naïve female virgin C57BL/6 mice of 8-12 weeks of age were obtained from a locally maintained colony (University of York, York, UK). Animals were housed under pathogen-free conditions under a 12 h light/dark cycle with free access to food and water. Mice were killed by carbon dioxide asphyxiation followed by cervical dislocation. Lower FRT tissue (vagina and cervix) was dissected and kept in sterile 1× PBS. Total blood was taken by cardiac puncture after verification of death and collected into tubes coated with heparin (Sigma-Aldrich, Gillingham, UK). Cervico-vaginal washings were taken from mice anaesthetized with isofluorane (Abbot, Maidenhead, UK) using sterile 1× PBS containing 0.5× Complete Protease Cocktail Inhibitor (Roche, Mannheim, Germany). A Microman® Precision Microliter pipette in conjunction with rounded-tip capillary pistons (Gilson, Luton, UK) was used for sample collection. All animal samples were kept on ice until subsequent use.

### Tissue staining

Vaginal smears and frozen vaginal tissue sections were stained with Mayer's hematoxylin (Sigma-Aldrich, Gillingham, UK) and counterstained with eosin Y (Sigma-Aldrich, Gillingham, UK) solution. The slides were equilibrated in increasing concentrations of ethanol solutions. Cells sorted by FACS were fixed with methanol and placed in Giemsa stain (VWR International, Lutterworth, UK) solution. Incubation was carried out for 20-40 minutes and the microscopy glass slides were thoroughly rinsed under cool running tap water. Stained slides were finally mounted with DePeX mounting medium (VWR International, Lutterworth, UK). Light microscopy was performed with an Axioplan microscope (Zeiss, Jena, Germany) in conjunction with the MagnaFire™ SP software v.2.1B (Olympus, Melville, USA).

### Assessment of estrous cycle stage

To allow proper cycling, the mice were housed in cages containing male soil and they were allowed to undergo at least two consecutive normal cycles before experimental work proceeded. Estrous cycle stage and cycle progress was conventionally assessed by vaginal smears taken each day between 8-10am. For this, mice were briefly anesthetized with isoflurane (Abbott, Maidenhead, UK). Once the animals were in a light sleep they were held upside-down at the base of their tail. Cervico-vaginal washes were taken by pipetting 30 µl of 1× sterile PBS up-and-down the vaginal cavity 10 times. This process was repeated thrice using a Microman® Precision Micoliter pipette in conjunction with rounded-tip capillary pistons (Gilson, Luton, UK). To avoid protein degradation 2 µl of 25× Complete Protease Inhibitor Cocktail (Roche, Mannheim, Germany) were added to the samples. A 2µl aliquot of the washings was smeared onto microscopy glass slides. Vaginal smears were air-dried, stained following a standard H&E protocol and cell composition was assessed thereafter with an Axioplan microscope (Zeiss, Germany).

### Assessment of endogenous cervico-vaginal IgG

After preparation of vaginal smears, cervico-vaginal washings were spun at 24,000xg for 10 min at 4°C. Sample collection was performed during each cycle stage every 24 hours so that endogenous mouse IgG, which had accumulated in this discrete time window, could be detected in the samples using an IgG-specific ELISA kit (Mabtech, Nacka Strand, Sweden), as per manufacturer's instructions with some modifications. Briefly, instead of using the supplied alkaline phosphatase (ALP)-conjugated goat anti-mouse IgG detection antibody included in the kit, a biotinylated version of the same detection antibody was used (Mabtech, Nacka Strand, Sweden). As an additional step, streptavidin-HRP (R&D Systems, Minneapolis, USA) was added to the ELISA plates for 30 minutes followed by incubation with 3,3′,5,5′-tetramethylbenzidine (TMB) substrate solution (Thermo Scientific, Rockford, USA). Color development was stopped after 10 minutes with 1 N H_2_SO_4_ and plates were read at 450 nm with a tunable VersaMax microplate reader in conjunction with the SoftMax Pro v.5.3 software (Molecular Devices, Sunnyvale, USA).

### Flow cytometry and FACS

Dissected lower FRTs (vagina and cervix) were minced and incubated in sterile RPMI-1640 medium (Dutch modification) (Sigma-Aldrich, Gillingham, UK) containing 0.5 mg/ml Liberase TL Research Grade (Roche, Mannheim, Germany) and 0.125 U DNase (Sigma-Aldrich, Gillingham, UK). Tissue samples were incubated for 1 h at 37°C at 220 rpm. Digested tissues were thoroughly vortexed and pipetted up and down using a 25 ml pipette. The tissue suspension was passed through a 70 µm cell strainer. A syringe was used to further break down undigested tissue clumps. The cell strainer was washed with 10 ml sterile RPMI-1640 medium and the single cell suspensions were spun at 300xg and re-suspended in 1× PBS. Blood samples were treated ×3 with Gey's solution and washed after each treatment with 1× PBS. The samples were spun each time at 300xg and finally re-suspended in 1× PBS.

FRT and blood cell suspensions were incubated for 15 minutes with 10 µg/ml of a CD16/32 antibody (Fc block) (eBioscience, Hatfield, UK). Samples were spun at 300xg and washed twice in 1× PBS. FRT cell suspensions were stained with following monoclonal antibodies; anti-mouse CD45.2- APC (clone 104), anti-mouse CD11c-PE-Cy7 (clone N418) (Biolegend, London, UK), anti-mouse CD11b-eFluor™ 450 (clone M1/70) (eBioscience, Hatfield, UK), anti-mouse lymphocyte antigen 6, locus C (Ly6C)-FITC (clone Al-21), anti-mouse lymphocyte antigen 6, locus G (Ly6G)-PE (clone 1A8) (BD Pharmingen, Oxford, UK) and fixable viability dye eFluor® 780 (eBioscience, Hatfield, UK). Blood cell suspensions were stained with anti-mouse granulocyte differentiation antigen (Gr)-1-PE (clone RB6-8C5) (BD Pharmingen, Oxford, UK) and anti-mouse CD11b-APC (clone M1/70) (eBioscience, Hatfield, UK). The appropriate isotype controls were included in the analyses. The final protein concentration of each antibody was 1 µg/ml. Incubation with primary antibodies was performed for 30 minutes followed by washing in 1× PBS. Samples were fixed with 2% paraformaldehyde (PFA) for 10 minutes and washed with 1× PBS. Flow cytometry was performed using a Cyan™ flow cytometer (Beckman Coulter, High Wycombe, UK) and data were analyzed with the Summit v.4.3 software (Beckman Coulter, High Wycombe, UK).

For FACS, cells were stained as described, with exclusion of the viability dye and 4-10 µg/ml of each antibody were used. FACS was performed with a MoFlo™ XDP cell sorter (Beckman Coulter, High Wycombe, UK) and data analysis was performed as described for flow cytometry experiments. Sorted cells were put onto glass slides using a cytospin (Thermo Scientific, Loughborough, UK). Samples were air-dried and stained with Giemsa.

### Immunohistology

Dissected lower FRTs were separated into vagina and cervix. Tissues were embedded in O.C.T. compound (Sakura Finetek, Thatcham, UK), slowly frozen on dry ice and subjected to serial cryo-sectioning. 8-10 µm thick frozen tissue sections were collected on poly-L-lysine-coated glass slides (VWR International, Lutterworth, UK) and air-dried. The tissue sections were fixed in acetone for 5 minutes and washed in 1× PBS with 0.05% BSA (wash buffer) followed by blocking in wash buffer containing 5% of rat serum and 10 µg/ml of a CD16/32 antibody (Fc block) (eBioscience, Hatfield, UK). Blocking was performed for 30 minutes. The tissue sections were washed thereafter in wash buffer and an Avidin/Biotin blocking kit (Invitrogen, Paisley, UK) was used, as per manufacturer's instructions. 2.5 µg/ml of biotinylated anti-mouse Gr-1 (clone RB6-8C5) (eBioscience, Hatfield, UK) and an anti-mouse F4/80 (clone BM8) (eBioscience, Hatfield, UK) or the appropriate isotype controls (eBioscience, Hatfield, UK) were added to the tissue sections. Incubation with the primary antibody was carried out for 45 minutes. The tissue sections were washed in wash buffer and streptavidin Alexa® Fluor 546 or streptavidin Alexa® Fluor 488 (Invitrogen, Paisley, UK) was added for 30 minutes. Subsequently, the sections were washed in wash buffer followed by a wash in 1× PBS. Tissue sections were counterstained with 1 µg/ml DAPI (Invitrogen, Paisley, UK) for 5 minutes. The slides were mounted with ProLong® Gold antifade reagent (Invitrogen, Paisley, UK) and subjected to confocal microscopy using a Zeiss LSM 510 meta microscope on an Axiovert 200 M. Images were analyzed using the Zeiss LSM Image Browser version 4.2.0.121. Quantification of imaging data was performed using the Image J software version 1.45. Data were analyzed by pixel count/unit area.

### Neutrophil depletion

Naïve virgin 8-12 week-old female C57BL/6 mice were screened for estrous cycle stage. Mice in each cycle stage were allocated to treatment groups and injected i.p. with either 250 µg of the Ly6G^+^ cell-depleting antibody clone 1A8 or the isotype control clone 2A3 (BioXCell, West Lebanon, USA). Endotoxins were removed from antibodies prior to injection with the Detoxi-Gel™ Endotoxin Removal Gel packed into columns (Thermo Scientific, Rockford, USA), as per manufacturer's instructions. The dose and effectiveness of 1A8 used in these studies was independently assessed [61]. Cervico-vaginal washings were taken 24 and 48h post-injection for assessment of estrous cycle stage and for analysis by mouse IgG-specific ELISA. Total blood and lower FRT tissue (vagina and cervix) were taken 48h after injection. Blood samples were analyzed by flow cytometry and tissue sections were subjected to fluorescence microscopy and H&E staining.

### Statistical analysis

Statistical analysis was performed using repeated measures ANOVA with Tukey's post test for paired sampling with more than 3 experimental groups, and unpaired t test for non-matched samples with less than 3 experimental groups. p≤0.05 was considered significant. All statistical analyses were performed with the GraphPad Prism5 software.

## Supporting Information

Figure S1 **Gating strategy for neutrophils and monocytes in the lower mouse FRT (vagina + cervix).** Tissue was taken from naïve virgin C57BL/6 female mice (8-12 weeks old) and prepared for FACS staining as described. Dead cells were excluded with a viability dye **(Step 1)**. Live cells were gated onto a forward scatter (x-axis) versus side scatter (y-axis) plot which further excluded cell debris and aggregates **(Step 2)**. CD45.2 was then used to select viable and single hematopoietic cells **(Step 3)** which were further discriminated between CD11b- and CD11c-expressing immune cells. A region was drawn around CD11b^+^ CD11c^−^ hematopoietic cells as the cells of interest **(Step 4)**. Viable and single hematopoietic cells which express CD11b but not CD11c were gated onto a Ly6C (x-axis) versus Ly6G (y-axis) plot **(Step 5)**. This step allowed distinguishing neutrophils (CD45.2^+^ CD11b^+^ CD11c^−^ Ly6C^+^ Ly6G^+^) from monocytes (CD45.2^+^ CD11b^+^ CD11c^−^ Ly6C^+^ Ly6G^−^). Each cell population was then gated onto a forward scatter (x-axis) versus side scatter (y-axis) plot to determine size and granularity **(Step 6)**.
(TIF)

Figure S2 **Assessment of systemic and local Ly6G depletion.** **(A)** Blood samples were taken from naϊve virgin C57BL/6 females (8-12 weeks old) treated with either mAb 2A3 or the isotype control 2A3, stained for Gr-1 (PE) and CD11b (APC) and analyzed by flow cytometry. **(B)** Flow cytometry data are derived from one experiment (n = 20) and were analyzed using unpaired t test. Error bars represent the mean±SEM (***, p≤0.001). **(C)** Vaginal tissue was taken from naïve virgin C57BL/6 female mice (8-12 weeks old) treated during PE with mAb 1A8 (n = 5) or control mAb 2A3 (n = 5) and stained with DAPI (blue) and for Gr-1 (green) and F4/80 (red). Isotype controls for each cell marker were included for the analysis. Representative images are shown. Images were taken with 200× magnification. The scale bar represents 50 µm. *, lumen; **, lamina propria; thick line, basal membrane; thin line; epithelial cell-lumen border. Data represent a single optical slice. **(D and E)** Quantitation of images (n = 15) was performed for abundance of Gr-1^+^ cells **(D)** and F4/80^+^ cells **(E)** with the Image J software. Quantitative data are derived from one experiment and were analyzed with unpaired t test. The mean±SEM is shown (n.s., non-significant; ***, p≤0.001).
(TIF)
