# Supplementary figures and images for: Cervico-Vaginal Immunoglobulin G Levels Increase Post-Ovulation Independently of Neutrophils
Source: PLoS One. 2014 Dec 5;9(12):e114824. doi: 10.1371/journal.pone.0114824 (PMC4257712; doi:10.1371/journal.pone.0114824)

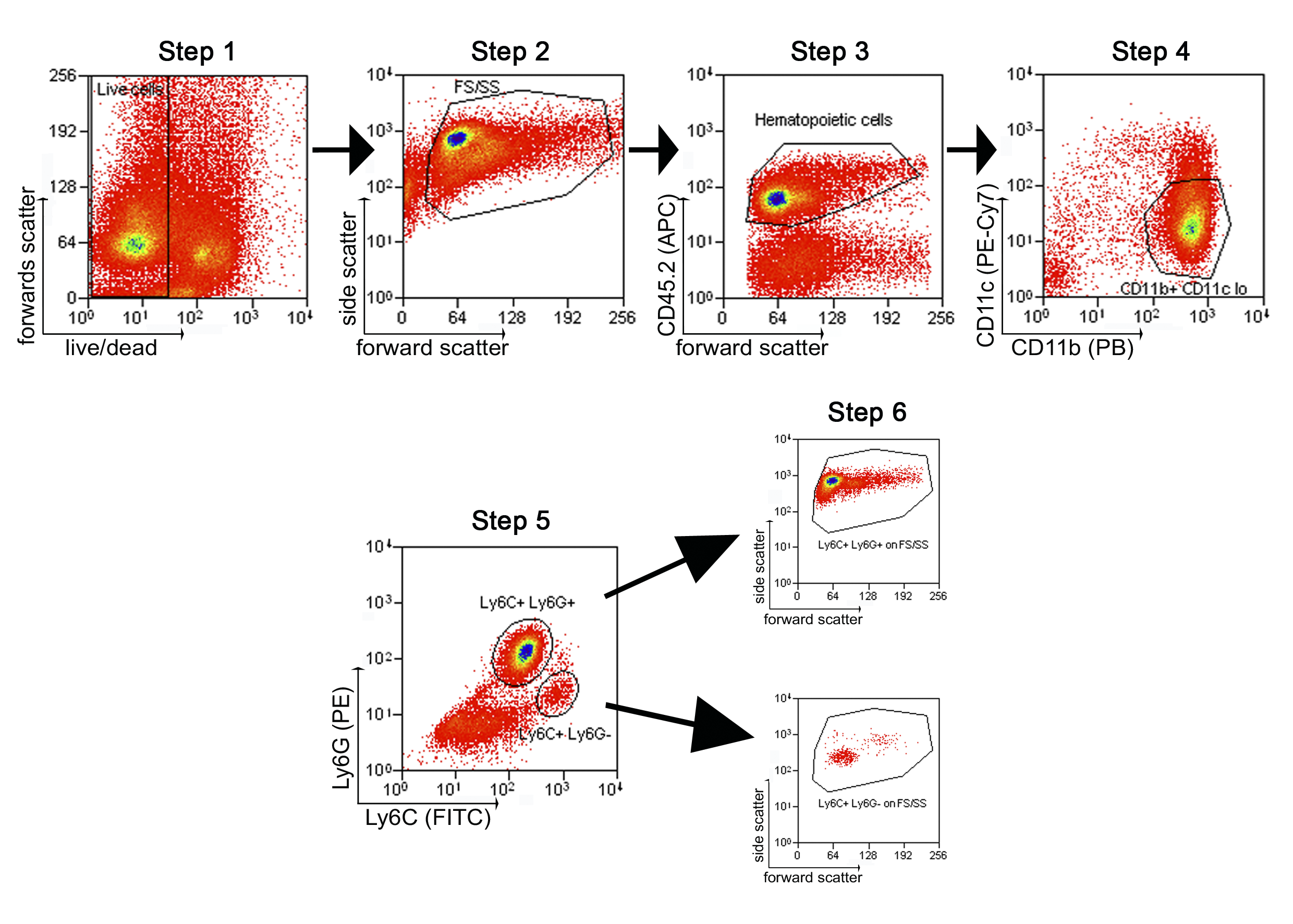

Supplement: Figure S1 — Gating strategy for neutrophils and monocytes in the lower mouse FRT (vagina + cervix). Tissue was taken from naïve virgin C57BL/6 female mice (8-12 weeks old) and prepared for FACS staining as described. Dead cells were excluded with a viability dye (Step 1). Live cells were gated onto a forward scatter (x-axis) versus side scatter (y-axis) plot which further excluded cell debris and aggregates (Step 2). CD45.2 was then used to select viable and single hematopoietic cells (Step 3) which were further discriminated between CD11b- and CD11c-expressing immune cells. A region was drawn around CD11b+ CD11c− hematopoietic cells as the cells of interest (Step 4). Viable and single hematopoietic cells which express CD11b but not CD11c were gated onto a Ly6C (x-axis) versus Ly6G (y-axis) plot (Step 5). This step allowed distinguishing neutrophils (CD45.2+ CD11b+ CD11c− Ly6C+ Ly6G+) from monocytes (CD45.2+ CD11b+ CD11c− Ly6C+ Ly6G−). Each cell population was then gated onto a forward scatter (x-axis) versus side scatter (y-axis) plot to determine size and granularity (Step 6). (TIF) [file pone.0114824.s001.tif]

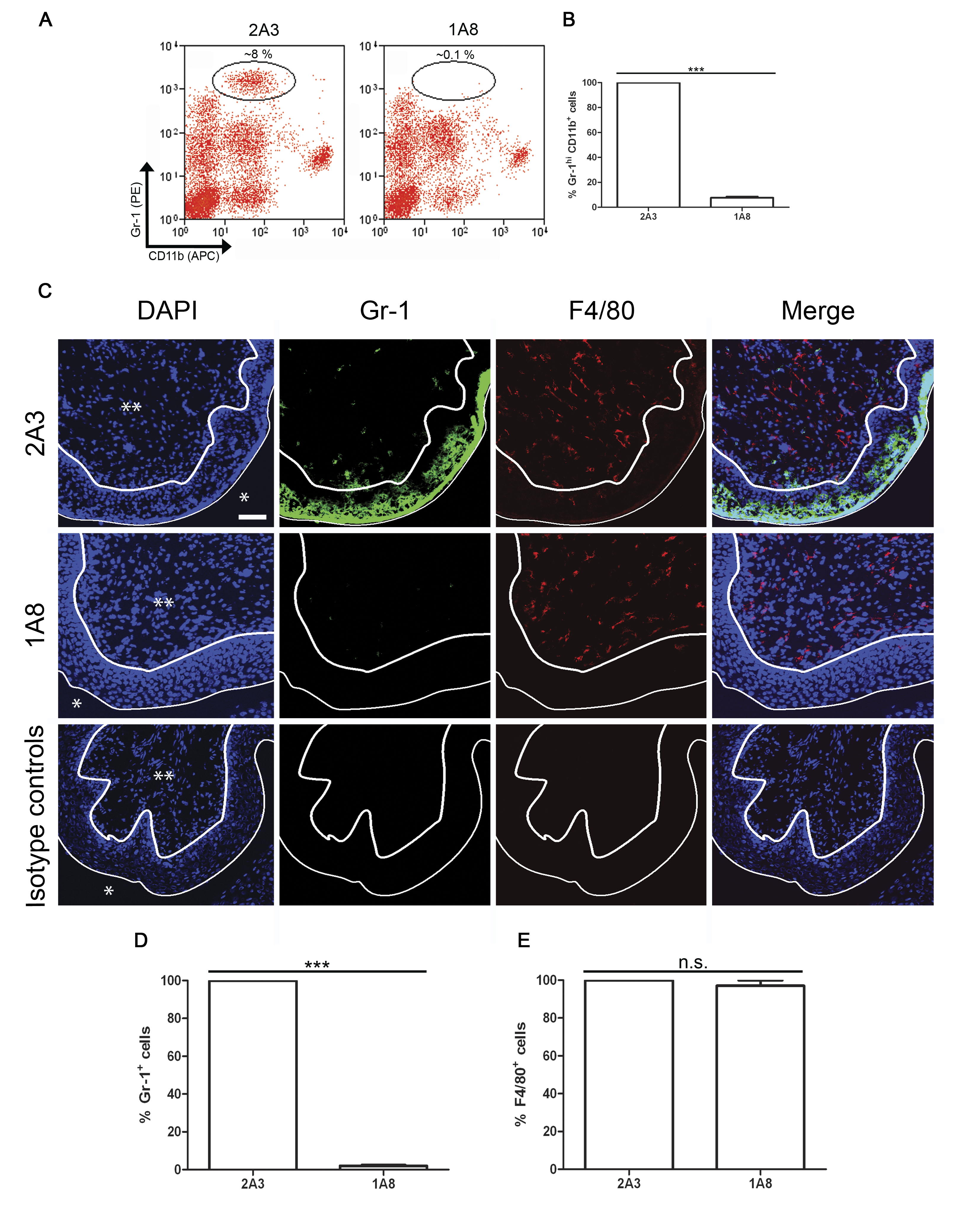

Supplement: Figure S2 — Assessment of systemic and local Ly6G depletion. (A) Blood samples were taken from naϊve virgin C57BL/6 females (8-12 weeks old) treated with either mAb 2A3 or the isotype control 2A3, stained for Gr-1 (PE) and CD11b (APC) and analyzed by flow cytometry. (B) Flow cytometry data are derived from one experiment (n = 20) and were analyzed using unpaired t test. Error bars represent the mean±SEM (***, p≤0.001). (C) Vaginal tissue was taken from naïve virgin C57BL/6 female mice (8-12 weeks old) treated during PE with mAb 1A8 (n = 5) or control mAb 2A3 (n = 5) and stained with DAPI (blue) and for Gr-1 (green) and F4/80 (red). Isotype controls for each cell marker were included for the analysis. Representative images are shown. Images were taken with 200× magnification. The scale bar represents 50 µm. *, lumen; **, lamina propria; thick line, basal membrane; thin line; epithelial cell-lumen border. Data represent a single optical slice. (D and E) Quantitation of images (n = 15) was performed for abundance of Gr-1+ cells (D) and F4/80+ cells (E) with the Image J software. Quantitative data are derived from one experiment and were analyzed with unpaired t test. The mean±SEM is shown (n.s., non-significant; ***, p≤0.001). (TIF) [file pone.0114824.s002.tif]
